# AAPM MEDICAL PHYSICS PRACTICE GUIDELINE 2.b.: Commissioning and quality assurance of X‐ray‐based image‐guided radiotherapy systems

**DOI:** 10.1002/acm2.13346

**Published:** 2021-07-16

**Authors:** Steven P. McCullough, Hassaan Alkhatib, Kyle J. Antes, Sarah Castillo, Jonas D. Fontenot, Andrew R Jensen, Jason Matney, Arthur J. Olch

**Affiliations:** ^1^ Highlands Oncology Group Fayetteville AR USA; ^2^ South Carolina Oncology Associates Columbia SC USA; ^3^ Baylor Scott and White Healthcare Irving TX USA; ^4^ Northside Hospital Atlanta GA USA; ^5^ Mary Bird Perkins Cancer Center Baton, Rouge LA USA; ^6^ Radformation New York NY USA; ^7^ UC Davis Medical Center Sacramento CA USA; ^8^ Keck School of Medicine University of Southern California Los Angeles CA USA; ^9^ Children’s Hospital of Los Angeles Los Angeles CA USA

**Keywords:** AAPM, IGRT QA, image guidance, MPPG, radiotherapy quality assurance

## Abstract

The American Association of Physicists in Medicine (AAPM) is a nonprofit professional society whose primary purposes are to advance the science, education, and professional practice of medical physics. The AAPM has more than 8000 members and is the principal organization of medical physicists in the United States. The AAPM will periodically define new practice guidelines for medical physics practice to help advance the science of medical physics and to improve the quality of service to patients throughout the United States. Existing medical physics practice guidelines will be reviewed for the purpose of revision or renewal, as appropriate, on their fifth anniversary or sooner. Each medical physics practice guideline represents a policy statement by the AAPM, has undergone a thorough consensus process in which it has been subjected to extensive review, and requires the approval of the Professional Council. The medical physics practice guidelines recognize that the safe and effective use of diagnostic and therapeutic radiology requires specific training, skills, and techniques, as described in each document. Reproduction or modification of the published practice guidelines and technical standards by those entities not providing these services is not authorized.

## 1 INTRODUCTION

Image‐guided radiation therapy (IGRT), in its many forms, is an important tool in improving the effectiveness of clinical radiation oncology. IGRT involves the use of patient images to localize and reposition the patient or delivery system prior to treatment to ensure that the therapeutic beam is correctly directed toward the target. IGRT imaging strategies have utilized X‐rays, ultrasound, and other means. In particular, IGRT has been most commonly facilitated using X‐rays, beginning with the use of megavoltage (MV) portal and/or orthogonal setup images some decades ago. These images provide a means of evaluating the position of the treatment isocenter and field edges relative to the patient's position. Due to the poor low‐contrast resolution of MV images, bony anatomy may be taken as a surrogate of the target volume, which is frequently soft tissue equivalent and not clearly visible within the image. However, as many studies have shown, the target volume can exhibit a different relative location to bony anatomy than expected. One solution to clinical scenarios in which improved soft tissue targeting is desired was the introduction of in‐room kilovoltage (kV) imaging systems. Such systems have included computed tomography (CT) scanners located within the treatment room (e.g., “CT‐on‐rails”) and kV imaging systems affixed to the floor/ceiling or to the linear accelerator gantry itself. These systems have provided improved the low‐contrast localization of soft tissue targets and—in the case of in‐room CT scanners and gantry‐mounted imaging systems—allowed for the acquisition of pretreatment volumetric images.

The specific choice and application of the IGRT strategy depend on the complexity and requirements of the treatment in question. It may serve as an enhancement to an established technique (as in the case of three‐dimensional conformal radiation therapy or intensity‐modulated radiation therapy) or as a necessary and critical component of the treatment process (as in the case of stereotactic body radiation therapy^1^). Currently, IGRT strategies are being used more than ever before, and various forms of IGRT have been, are, and will continue to be important tools in radiation therapy. The report of AAPM Task Group 104^2^ provides an instructive overview of the various uses of X‐ray imaging in radiation therapy.

As the clinical treatment process continues to rely more heavily on IGRT strategies, the Qualified Medical Physicist (QMP) is under increasing pressure to maintain patient safety and treatment quality through quality assurance programs that address the image acquisition and formation systems used in IGRT and that are in step with the rapid pace of imaging technological development. Many clinical practice environments now utilize treatment delivery systems with one or more IGRT systems that fall under the responsibility of the QMP. A variety of guidance documents and task group reports have been issued that include additional recommendations for commissioning and quality assurance of IGRT or planning CT systems.^2^, ^3^, ^4^, ^5^, ^6^, ^7^, ^8^, ^9^, ^10^ However, these reports do not clearly delineate *best* practice from *minimum* practice standards.

### 1.1 Goals and rationale

This document is part of a series of medical physics practice guidelines commissioned by the American Association of Physicists in Medicine (AAPM) intended to succinctly state the minimum acceptable standards for various aspects of clinical medical physics. This report is the first revision of MPPG 2 first published in 2014. While the implementation of robust and comprehensive quality assurance programs recommended in other reports from the AAPM is encouraged, the purpose of this particular report is to describe the minimum acceptable practice standards for the commissioning and quality assurance of X‐ray‐based image guidance systems utilized in radiotherapy. This document is not intended to replace or revise previous AAPM Task Group Reports, but to assist the QMP in establishing and maintaining a safe and effective IGRT program by providing an overview of the minimum requirements and needs of X‐ray‐based IGRT systems. Indeed, the reader is referred to the appropriate technical reference documents or task group reports in instances when additional recommendations beyond minimum practice guidelines are desired.^1^, ^2^, ^3^, ^4^, ^5^, ^6^, ^7^, ^8^, ^9^, ^10^ Finally, the standards and procedures described in this document are applicable to the imaging guidance system insofar as its resulting images are used to position the patient and/or localize the target volume. The use of IGRT system hardware and software for other purposes, such as the use of the IGRT images for treatment plan dose calculation, are beyond the scope of this report. Technologies covered by these guidelines include:

1. Gantry‐mounted two‐dimensional MV imaging systems.
2. Gantry‐mounted three‐dimensional MV imaging systems.
3. Gantry‐mounted two‐dimensional kV imaging systems.
4. Gantry‐mounted three‐dimensional kV imaging systems.
5. Room‐mounted two‐dimensional kV imaging systems.
6. Room‐mounted three‐dimensional kV imaging systems.

In this context, “gantry‐mounted” imaging systems are those in which the mechanical movement of the imaging hardware is coupled with the mechanical movement of the treatment delivery device (e.g., Varian/Elekta/Siemens on‐board kV imaging systems, electronic portal MV imaging systems, and TomoTherapy megavoltage CT). “Room‐mounted” systems include all imaging systems not coupled with the treatment delivery device (e.g., ExacTrac, CyberKnife, and in‐room CT). Fluoroscopy modes are also within the scope of this report.

### 1.2 Intended users

The intended users of this report are QMPs who seek to understand the technical requirements of clinical implementation and quality assurance of a safe IGRT practice, and administrators interested in the resources required for IGRT.

This committee recommends that practicing medical physicists systematically evaluate potential failures in workflow and process. Task Group 100 of the AAPM has developed a framework for designing quality management (QM) activities based upon estimating probabilities of identified failures and their clinical implications. This report will not specifically outline Task Group 100 procedures, but rather recommends that the practicing physicist reference that publication, its methodology, and nomenclature in the development of a QM program for X‐ray image guidance utilizing this publication as a basis for minimum practice standards.

## 2 DEFINITIONS AND ABBREVIATIONS

CBCT – cone‐beam computed tomography.

IGRT – image‐guided radiotherapy.

kV ‐ kilovoltage.

MV ‐ megavoltage.

OIS ‐ oncology information system.

QA ‐ quality assurance.

TPS ‐ treatment planning system.

## 3 STAFF QUALIFICATIONS AND RESPONSIBILITIES

Implementation of a successful IGRT program requires contributions from each member of the treatment team. Recommendations for staff qualifications and responsibilities are consistent with those described by the ACR‐ASTRO practice guideline for clinical use of IGRT.^9^

### 3.1 Medical physicist

The qualified medical physicist (QMP) must be competent to practice independently in the subfield of medical physics (such as therapeutic and diagnostic medical physics) appropriate to the assigned tasks. The individual must be certified by the American Board of Radiology, American Board of Medical Physicists, and the Canadian College of Medical Physicists.

Responsibilities of the qualified medical physicist in an IGRT program include:

1. Acceptance testing and commissioning.
2. Implementing and managing a quality assurance program.
3. Developing and implementing standard operating procedures (including imaging protocols and repositioning thresholds).

The QMP may be assisted by medical physics residents or medical physicist assistants with these responsibilities provided (a) these individuals have been appropriately trained to perform the assigned tasks and (b) the QMP provides general supervision of all work performed. A qualified diagnostic physicist could work collaboratively with the qualified therapy physicist to implement and conduct a successful IGRT QA program but ultimately program implementation is the responsibility of the qualified therapy physicist.

### 3.2 Radiation Oncologist

The radiation oncologist should meet qualifications outlined in the ACR‐ASTRO practice guideline for clinical use of IGRT.^11^ In short, the responsibilities of the radiation oncologist in an IGRT program include:

1. Specifying patient positioning procedures.
2. Specifying imaging modalities and frequencies.
3. Identifying registration targets and repositioning thresholds.
4. Conducting a timely review of clinical IGRT images.
5. Conducting regular reviews of the IGRT program.
6. Implementing and managing a quality assurance program.
7. Developing and implementing standard operating procedures (including imaging protocols and repositioning thresholds).

### 3.3 Medical dosimetrist

The medical dosimetrist should meet the qualifications outlined in the *Scope of Practice of a Medical Dosimetrist* approved by the Board of Directors of the American Association of Medical Dosimetrists.^12^ Responsibilities of the medical dosimetrist or treatment planner in an IGRT program include:

1. Creating and transferring to the OIS all patient‐specific data necessary for IGRT implementation.

### 3.4 Radiation therapist

The radiation therapist should meet the qualifications outlined in *Radiation Therapy Practice Standards* issued by the American Society of Radiologic Technologists.^13^ Responsibilities of the radiation therapist in an IGRT program include:

1. Understanding the use of positioning devices in IGRT.
2. Preparing the IGRT system for the acquisition of patient‐specific positioning verification images.
3. Implementing the IGRT imaging protocol under the supervision of the radiation oncologist and medical physicist.
4. Acquiring positioning verification images for review by the radiation therapist or radiation oncologist.
5. Assisting in periodic review of the stability of the IGRT system (e.g., daily QA).

### 3.5 Information technology specialist

It is important that each facility identify an individual that is responsible for providing and maintaining resources necessary for storing, archiving, and retrieving images generated during IGRT. This may be accomplished by a dedicated Information Specialist or duties assigned to another team member.

## 4 IMPLEMENTATION GUIDELINES

### 4.1 Minimum required resources and equipment

#### 4.1.1 Staffing

Approximate time requirements needed for implementation, maintenance, and quality assurance of each IGRT program type (per each IGRT system) are provided below. Estimates are provided as general reference values only and are not intended to justify site‐specific staffing models or physics time for specific billing codes. "Acceptance/commissioning" includes all activities needed for IGRT program implementation, including documentation. "Documentation" refers to the creation of a formal commissioning report and drafting of policies and procedures specific to clinical use and routine quality assurance of IGRT (including creating QA forms and templates). "Ongoing support" includes all activities needed for the maintenance of an established IGRT program (e.g., routine quality assurance, imaging protocol development and review, troubleshooting, upgrades, and service and repairs).

1. Two‐dimensional MV imaging systems.
  - Acceptance/Commissioning/Documentation: 8–12 hours.
  - Ongoing support: 8–16 hours annually.
2. Three‐dimensional MV imaging systems.
  - Acceptance/Commissioning/Documentation: 8–20 hours.
  - Ongoing support: 8–16 hours annually.
3. Two‐dimensional kV imaging systems.
  - Acceptance/Commissioning/Documentation: 8–12 hours.
  - Ongoing support: 8–16 hours annually.
4. Three‐dimensional kV imaging systems.
  - Acceptance/Commissioning/Documentation: 8–20 hours.
  - Ongoing support: 8–16 hours annually.

### 4.2 Equipment

Quality assurance phantoms and tools must provide reliable values of the measured parameters and can be used to judge whether tolerance criteria have been achieved. In many cases, manufacturers of IGRT systems provide quality assurance phantoms that can be used for quality assurance purposes. In‐house and commercial phantoms specifically designed for IGRT are also available and, when coupled with automated image analysis tools, may improve efficiency. At a minimum, quality assurance tools must be capable of assessing the following IGRT characteristics:

1. Image quality (i.e., contrast, Resolution, Uniformity).
2. Spatial accuracy (scaling).
3. Congruence of imaging and treatment isocenters.
4. Accuracy of registration/table movements.
5. Imaging dose.

### 4.3 Staff training

Training for the operation of the IGRT system must be provided. The IGRT system vendor typically provides on‐site training to the physicist and therapists for use of the equipment. Prior to the initial use of IGRT, the treatment team should meet to discuss staff responsibilities, clinical goals, and process workflows. The physicist should also review the image acquisition procedures with the therapists and radiation oncologists. Consultation with a QMP certified in diagnostic imaging to develop optimized data acquisition and image formation protocols is advantageous and recommended. In general, IGRT training will require additional dedicated staff time that is not included in the estimated time requirements of Section 4.a.1. In addition to initial training, it is important that each facility develop a periodic training review program to ensure competency on current systems and augment with training for system upgrades/changes. Formal training of new staff not present at initial training should be conducted.

### 4.4 Process descriptions

Example procedures for each of the tests recommended in Table 1 are described below. The approximate time needed to complete each procedure is noted in parenthesis following the process description. In some cases, customer acceptance procedures provided by the equipment vendor satisfactorily meet the stated practice standards; however, it is the responsibility of the QMP to judge the adequacy and completeness of all measurements needed for use of a particular IGRT system. It is important to note that these are only example procedures, and a variety of methods may be used to complete the recommended tests. Certain commercially available products are referred to by name. These references are for informational purposes only and imply neither endorsement by the AAPM nor that these are the best or the only products available for the stated purpose.

**TABLE 1:** Recommended minimum practices for commissioning and QA of an IGRT system.

Acceptance testing and commissioning	
Procedure	
Customer acceptance procedures	
TPS integration	
OIS integration	
Establish routine QA baselines	
QA documentation	
Routine quality assurance	
Daily or day of special procedure	
Procedure	Tolerance
Safety/interlocks	Functional
Imaging‐treatment isocenter coincidence and table positioning composite (SRS only)	1 mm
Imaging‐treatment isocenter coincidence (lasers as treatment reference)	2 mm
Table positioning/repositioning	2 mm
Monthly	
Procedure	Tolerance
Imaging‐treatment isocenter coincidence (MV image as reference)	2 mm
Semi‐annually	
Procedure	Tolerance
Image scaling	2 mm
Annually	
Procedure	Tolerance
Gating Interlock	Functional
Imaging dose	
2D MV	± 1 cGy of the baseline value
2D kV (static imaging mode)	± 3 mGy of the baseline value
2D kV (fluoroscopy mode)	± 1 cGy/min of the baseline value
All 3D imaging modes	± 1 cGy of the baseline value
Image quality	
2D (spatial resolution, contrast)	At least baseline value
3D (uniformity, spatial resolution, contrast)	At least baseline value
Upgrade/Repair/Service	
Manufacturer recommended testing	As recommended
Verify / Reestablish QA baselines (as appropriate)	As needed post‐change

SRS, stereotactic radiosurgery; SBRT, stereotactic body radiation therapy.

#### 4.4.1 Customer acceptance procedures (all systems)

The QMP must provide direct supervision during the acceptance testing process.^14^ Customer acceptance test procedures are intended to ensure that the imaging equipment satisfies the performance requirements stated in the purchase agreement. In some cases, measurements completed as part of the acceptance procedures may also serve as components in establishing the routine quality assurance program. The vendor must demonstrate acceptable system performance. (Time: 4–8 hours).

#### 4.4.2 TPS configuration and connectivity (2D systems)

Digitally reconstructed radiographs (DRR) of test objects in various orientations are created with the treatment planning system and transferred (typically via DICOM interface) to the image guidance system. Proper display of the DRR image within the image guidance software must be ensured. (Time: 3–4 hours).

#### 4.4.3 TPS configuration and connectivity (3D systems)

Reference CT image sets of test objects in various orientations are imported into the treatment planning system. Contours are added and the images and structures are transferred (typically via DICOM interface) to the image guidance system. Proper display of the reference CT images and structures within the image guidance software must be ensured. (Time: 3–4 hours).

#### 4.4.4 OIS integration and connectivity (2D systems)

Setup fields created for a test patient within the oncology information system are properly recognized by the imaging hardware and software when loaded. Acquired images are then assigned to the correct patient, if applicable. (Time: 2–3 hours).

#### 4.4.5 OIS integration and connectivity (3D systems)

Volumetric IGRT image setup fields (CBCT, MVCT, CT‐on‐rails) created for a test patient within the oncology information system are properly loaded and recognized by the imaging hardware and software. Acquired images are assigned to the correct patient and are available for registration with the reference 3D image set. (Time: 2–3 hours).

#### 4.4.6 Routine QA baselines (all systems)

Measurements taken at the time of IGRT system commissioning, which characterizes IGRT system performance will serve as reference values for the routine QA program. See Table 1 for recommended QA tests requiring reference measurements. (Time: 2–3 hours).

#### 4.4.7 IGRT QA program documentation (all systems)

All acceptance and commissioning procedures and results must be contained within a formal report. Furthermore, a formal policy for routine IGRT QA programs and procedures for performing routine QA measurements must be developed. (Time: 4–8 hours).

#### 4.4.8 *Safety*/*interlocks (all systems)*

With image acquisition initiated, ensure beam termination occurs when the treatment room door is opened (if applicable) and when any termination keys are depressed. If images are to be acquired with the treatment room door open, then measurements and calculations of exposure should be performed at the treatment console to ensure safe operating conditions. Also, ensure that gantry rotation is terminated when touch guards are depressed. Verify that indicator lamps are illuminated during image acquisition. Systems with the capability to pause (beam hold) or terminate beam delivery based upon imaging results should be tested to ensure the beam hold or terminate functions as expected (Time: 20 minutes).

#### 4.4.9 Contrast (2D kV systems)

A phantom with low‐contrast objects is placed on the treatment table at the isocenter. A planar kV image is acquired using a reference technique determined at the time of acceptance testing. The window and level are adjusted to reference values determined at the time of acceptance testing. The number of visible disks is recorded, with more indicating better low‐contrast visibility. (Time: 15 minutes).

#### 4.4.10 Contrast (2D MV systems)

A phantom with low‐contrast objects is placed on the treatment table. A planar MV image is acquired using a reference technique determined at the time of acceptance testing. The window and level are adjusted to a reference value determined at the time of acceptance testing. The number of visible disks of largest diameter or frequency groups is recorded, with more indicating better low‐contrast visibility. (Time: 15 minutes).

#### 4.4.11 Contrast (3D systems)

An appropriate volumetric image quality phantom is positioned on the treatment table using the room lasers. A volumetric image is acquired using a reference technique determined at the time of acceptance testing. Either the difference in CT number of different materials within the phantom or the number of visible low‐contrast objects is recorded. (Time: 15 minutes).

#### 4.4.12 Spatial resolution (2D kV systems)

A phantom with high‐contrast objects is placed on the treatment table. A planar kV image is acquired using a reference technique determined at the time of acceptance testing. The number of frequency groups that are clearly distinguished is recorded, with more frequency groups indicating better spatial resolution. (Time: 15 minutes).

#### 4.4.13 Spatial resolution (2D MV systems)

A phantom with high‐contrast line groups is placed on the treatment table. A planar MV image is acquired using a reference technique determined at the time of acceptance testing. The number of visible line groups is recorded, with more indicating better spatial resolution. (Time: 15 minutes).

#### 4.4.14 Spatial resolution (3D systems)

An appropriate volumetric image quality phantom is positioned on the treatment table using the room lasers. A volumetric image is acquired using a reference technique determined at the time of acceptance testing. The number of frequency groups that are clearly distinguished is recorded, with more frequency groups indicating better spatial resolution. (Time: 15 minutes).

#### 4.4.15 Scaling (all systems)

A phantom of known dimensions is placed on the treatment table using the room lasers. A planar or volumetric image is acquired. Window and level are adjusted such that the phantom is clearly visible. The distance between two objects of known separation in the horizontal and vertical axes is recorded and compared with the known distance. For 3D imaging systems, scaling must be measured in all three dimensions. (Time: 10 minutes).

#### 4.4.16 Uniformity (3D systems)

An appropriate volumetric image quality phantom is positioned on the treatment table using the room lasers. A volumetric image is recorded. The average pixel value over a region of interest at multiple locations (e.g., center, 12 o'clock, and 3 o'clock) is recorded and compared. (Time: 15 minutes).

#### 4.4.17 *Imaging*‐*treatment isocenter coincidence (2D systems)*

A variety of methods may be used to verify the congruence of imaging and treatment isocenters. Most of these techniques require the alignment of a radiopaque marker (e.g., ball bearing, fiducial, or commercial device) to treatment isocenter via room lasers (assuming laser congruence was established within 1 mm) or by alignment to the center of an MV image (or orthogonal images). Orthogonal kV images are then acquired. The congruence of imaging and treatment isocenters is verified by comparing the position of the markers with the center of the kV images. (Time: 10–15 minutes).

#### 4.4.18 *Imaging*‐*treatment isocenter coincidence (3D systems)*

A variety of methods may be used to verify the congruence of imaging and treatment isocenters. Most of these techniques are usually coupled with the “table positioning/repositioning” test and begin with the positioning of a radiopaque marker (ball bearing, fiducial, or commercial device) on the treatment table with known offsets from the isocenter (offsets not required). Volumetric IGRT images are then acquired and registered with reference images. The recommended table shifts from the image registration software are recorded and applied, and the coincidence of the imaging and treatment isocenters is assessed by comparing the coincidence of the marker with the center position of an acquired MV portal image. (Time: 15 minutes).

#### 4.4.19 Table Positioning/Repositioning (all systems)

A phantom with radiopaque markers is positioned on the treatment table with known offsets from the isocenter. Images are acquired and registered with reference images. The recommended table shifts from the image registration software are recorded and applied. Proper functioning of the image registration software is verified by comparing the recommended table shifts with the known offsets, while the correct application of the shifts is assessed by comparing the position of the external markers on the phantom with the room lasers. (Time: 15 minutes).

#### 4.4.20 Imaging dose (3D systems)

Several different methods are currently used to characterize the dose from 3D IGRT systems. The traditional metric for dose from CT imaging, the computed tomography dose index (CTDI), has been applied to IGRT imaging. More recently, the AAPM Task Group 111^9^ report has introduced a new metric, the cumulative dose. These two different metrics use different measurement equipment and irradiation geometries to characterize the dose.

Measurement equipment used to measure CTDI includes a calibrated 100 mm long pencil ionization chamber and an appropriate phantom, one simulating a head and the other a large pelvis. For each imaging mode, the phantom that matches the mode's target anatomy is used. The phantom is positioned at the isocenter and the pencil chamber is positioned in the phantom. The radiation field length must be constrained to be less than the active length of the pencil ionization chamber. For each imaging mode, measurements are repeated for the central and peripheral phantom locations.

Measurement equipment used to measure the cumulative imaging dose includes a Farmer‐type ionization chamber calibrated in the appropriate energy range and several acrylic CTDI phantoms.

Measured imaging dose must be documented and its management should be approached to keep it as low as necessary to achieve clinically useful images. (Time: 15–60 minutes, depending on the number of techniques measured).

#### 4.4.21 Imaging dose (2D systems)

Imaging dose from 2D kV systems is most typically characterized using entrance surface air kerma (skin exposure). Measurement equipment used to measure the entrance air kerma includes a calibrated ionization chamber on the surface of a phantom (or an in‐air measurement can be made with a backscatter factor applied). A source–detector distance of 100 cm is set and the field size is set to cover the detector. A clinically relevant beam is delivered, and the air kerma rate is calculated for static and fluoroscopic imaging modes, respectively.

Measured imaging dose should be documented and its management should be approached to keep it as low as necessary to achieve clinically useful images. (Time: 15–60 minutes, depending on the number of techniques measured).

### 4.5 Continuing quality improvement

Ongoing review and audit of the IGRT program should occur at regular intervals. In particular, periodic review of clinical image registration, the use of appropriate imaging techniques/frequencies, adherence to stated QA programs, and revision of IGRT strategies based on pertinent changes in clinical practices should be assessed.

## 5 RECOMMENDATIONS

Recommended minimum practices for commissioning and QA of an IGRT system are shown in Table 1. Test frequencies and tolerance values were developed based on relevant AAPM Task Group reports and the experience of the MPPG members in relation to the stability and importance of each parameter to the IGRT process. Sample process descriptions are included in Section 5.c. The “baseline value” shown in the table refers to the IGRT system manufacturer's minimum performance standard stated in the customer acceptance procedure documentation. If unavailable or not specified, then “baseline value” can be taken as the value measured at the time of commissioning. For example, most IGRT system manufacturers have stated performance specifications for image quality and, in such cases; those may serve as the tolerance values for routine QA measurements of image quality. However, most IGRT system manufacturers do not have stated performance specifications for imaging dose and, in such cases, the imaging dose measured at the time of commissioning may serve as the baseline value to which future measurements are compared.

Evaluation of imaging‐treatment isocenter coincidence and positioning/repositioning is considered critical. While daily checks of these parameters are preferred, weekly checks are considered acceptable for IGRT systems used with standard fractionation schemes. For IGRT systems used for SRS/SBRT, daily QA testing frequency must also be required on days when procedures are scheduled.

The imaging dose from an IGRT system must be measured at least annually for at least one acquisition technique of each mode of clinical operation. Dose and image quality must be remeasured after service activities affecting a major service component, such as the replacement of the X‐ray tube. For example, a gantry‐mounted kV system used to acquire both 2D and 3D clinical images must have documented imaging doses for both 2D and 3D modes. If imaging dose is only measured for one acquisition technique, then the chosen technique should serve to provide the most conservative value (e.g., choose the acquisition technique that results in the greatest imaging dose). For 2D kV systems operated in fluoroscopy mode, the entrance air kerma rate should be measured for the technique expected to produce the highest value.

These recommendations must also be augmented with procedures required by state regulations (such as the measurement of X‐ray tube voltage accuracy, where applicable). Furthermore, IGRT systems with known recurring problems should be subjected to more frequent QA at the discretion of the QMP. Annual end‐to‐end tests are also an effective method of assessing overall IGRT system accuracy but are not required in this report.

## 6 CONCLUSIONS

IGRT is a powerful and increasingly essential component of clinical radiation oncology practice. Proper use and quality assurance of clinical IGRT systems are of critical importance to maximizing the benefits and minimizing the risks of the technology. The minimum technical requirements for managing a clinical IGRT program stated in this document will help to achieve a more uniform standard of practice that improves the safety and quality of care of patients for whom IGRT is needed.

## DISCLOSURE STATEMENT

The Chair of Task Group No. 335 ‐ MPPG 2.b ‐ Commissioning and quality assurance of X‐ray‐based image‐guided radiotherapy (TG335) has reviewed the required conflicts of interest statement on file for each member of TG335 and determined that the disclosure of potential conflicts of interest is an adequate management plan. Disclosures of potential Conflicts of Interest for each member of TG335 are found at the close of this document.

1. The members of TG335 listed below attest that they have no potential conflicts of interest related to the subject matter or materials presented in this document.

Steven P McCullough, PhD, Chair.

Hassaan Alkhatib, PhD.

Kyle Antes, MS.

Sarah Castillo, PhD.

Jonas Fontenot, PhD.

Jason Matney, MS.

Arthur J. Olch, PhD, FAAPM.

2. The members of TG335 listed below disclose the following potential conflict(s) of interest related to the subject matter or materials presented in this document.

Andrew R Jensen, MS, is an employee of Radformation and has Radformation stock options.

Approved by AAPM’s Executive Committee January 25, 2021.
